# A variant of *PSMD6* is associated with the therapeutic efficacy of oral antidiabetic drugs in Chinese type 2 diabetes patients

**DOI:** 10.1038/srep10701

**Published:** 2015-05-29

**Authors:** Miao Chen, Cheng Hu, Rong Zhang, Feng Jiang, Jie Wang, Danfeng Peng, Shanshan Tang, Xue Sun, Jing Yan, Shiyun Wang, Tao Wang, Yuqian Bao, Weiping Jia

**Affiliations:** 1 Shanghai Diabetes Institute, Department of Endocrinology and Metabolism, Shanghai Clinical Center for Diabetes, Shanghai Key Clinical Center for Metabolic Disease, Shanghai Key Laboratory of Diabetes Mellitus, Shanghai Jiao Tong University Affiliated Sixth People’s Hospital, 600 Yishan Road, Shanghai, China; 2 Shanghai Jiao Tong University Affiliated Sixth People’s Hospital South Campus, Shanghai, China

## Abstract

The *PSMD6* variant rs831571 has been identified as a susceptibility locus for type 2 diabetes mellitus (T2DM). This study aimed to investigate the association of this variant with therapeutic effects of oral antidiabetic drugs in Chinese T2DM patients. 209 newly diagnosed T2DM patients were randomly assigned to treatment with repaglinide or rosiglitazone for 48 weeks, and the therapeutic effects were compared. In the rosiglitazone cohort, rs831571 showed significant associations with fasting plasma glucose (FPG), 2-h glucose and decrement of glycated haemoglobin (HbA1c) levels after 24 weeks of treatment (*P = *0.0368, 0.0468 and 0.0247, respectively). The C allele was significantly associated with a better attainment of FPG at 24 and 32 weeks (*P* = 0.0172 and 0.0257, respectively). Survival analyses showed CC homozygotes were more likely to attain a standard FPG level (*P* = 0.0654). In the repaglinide cohort, rs831571 was significantly associated with decreased HbA1c levels after 24 weeks of treatment, the homeostatic model assessment of insulin resistance and fasting insulin level after 48 weeks of treatment with repaglinide (*P* = 0.0096, 0235 and 0.0212, respectively). In conclusion, we observed that the *PSMD6* variant rs831571 might be associated with the therapeutic effects of rosiglitazone and repaglinide in Chinese T2DM patients. However, these findings need to be confirmed in the future.

Type 2 diabetes mellitus (T2DM) is a chronic disease that is growing worldwide at an epidemic rate, and the huge cost of treatment for T2DM is a heavy public health burden. Despite the availability of various drug classes for the treatment of T2DM, the management of hyperglycaemia is not satisfactory. For all known efficacious interventions, some patients respond to treatment, whereas others do not. This divide has been partly attributed to the various clinical responses to the drugs among individuals. In addition, other factors such as age, gender, liver and renal function, severity of the disease, genetic factors involved in drug absorption, distribution, metabolism, and drug targ*et al*so play an important role in the observed individual variability^1^.

With the development of genome-wide association studies (GWASs), an increasing number of susceptibility loci for T2DM have been identified. A lot of T2DM susceptibility genes, such as *PAX4*, *KCNQ1*^2^,^3^ and so on, had been reported associated with pharmacogenomics effects of oral antidiabetic drugs including thiazolidinediones (TZDs) and glinides^4^. The single-nucleotide polymorphism (SNP) rs831571 in *PSMD6* gene has been identified as a novel susceptibility locus for T2DM in different ethnic populations^5^. The encoded protein of *PSMD6* gene is a subunit of the 26S proteasome, which is a member of the ubiquitin-proteasome system (UPS)^6^. UPS is the major cellular machinery responsible for both the recognition and degradation of proteins^7^, and has also been reported to have important roles in mediating the actions of insulin and insulin secretion^8^,^9^.

However, it remains unknown whether this SNP influences the therapeutic effects of oral antihyperglycaemic drugs. Thus, we chose newly diagnosed patients treated with either repaglinide or rosiglitazone to investigate the association of the *PSMD6* variant rs831571 with the therapeutic effects of these drugs.

## Results

Of the total 105 patients recruited in the rosiglitazone cohort, 93 completed the 48-week study. Of the twelve patients who were withdrawn from the study, one patient had elevated hepatic enzymes, 5 patients with inadequate control of blood glucose were excluded, and 6 patients were lost to follow-up. Genotyping was not performed one patient, and 92 patients were ultimately included in the statistical analysis. The CC, CT and TT genotype distribution of rs831571 among the rosiglitazone patients was 35, 47 and 10. Of the 104 total patients treated with repaglinide, 91 completed the entire study. Among the 13 patients who withdrew, 4 had a glycated haemoglobin (HbA1c) level ≥8% at two consecutive time points, and 9 patients were lost to follow-up. The CC, CT and TT genotype distribution of rs831571 among the repaglinide patients was 34, 50 and 7. The genotype distribution of *PSMD6* rs831571 was in agreement with Hardy–Weinberg equilibrium.

### Rosiglitazone cohort

The association between rs831571 and clinical parameters before and after rosiglitazone treatment is shown in Table 1. At baseline, there was no significant difference between the genotype groups with regard to all the clinical parameters. After 24 weeks of treatment with rosiglitazone, rs831571 was significantly associated with body mass index (BMI), fasting plasma glucose (FPG) and 2-h glucose (*P = *0.0309, 0.0368 and 0.0468, respectively). In addition, rs831571 also showed a significant association with reduced HbA1c levels after 24 weeks of treatment (*P = *0.0247). We also observed the trends towards associations between rs831571 with the homeostatic model assessment of beta cell function (HOMA-B) and its augmentation after 24 weeks of rosiglitazone treatment (*P = *0.0561 and 0.0547, respectively). Besides, we also found there were differences between different genotype in HbA1c levels after 24 weeks of treatment, the decrement of 2-h glucose after 24 weeks of treatment and HbA1c levels after 48 weeks of treatment (*P = *0.0233, 0.0364 and 0.0460, respectively).

We defined a FPG level of <6.1 mmol/L, a 2-h glucose of <7.8 mmol/L, and an HbA1c of <6.5% as attainment and used logistic regression analysis adjusted for age, gender, and BMI at baseline to estimate the genotype distributions between two groups with different responses to the drugs. We identified that the rs831571 C allele was significantly associated with a better attainment of FPG at the 24- and 32-week time points (*P = *0.0172 and 0.0257, OR (95%CI) = 2.358(1.164, 4.778) and 2.224(1.102, 4.489), respectively). Furthermore, we observed the trends towards associations between the rs831571 C allele and better attainment of FPG at 12 and 48 weeks (*P = *0.0680 and 0.0895, OR (95%CI) = 1.904(0.9533, 3.801) and 1.822(0.9115, 3.643), respectively).

We further adopted Cox regression model analysis to explore associations between rs831571 and the rate of attainment of target levels of FPG, 2-h glucose, and HbA1c. Survival analyses showed that after adjusting for age, gender, and BMI at baseline, rs831571 CC homozygotes were more likely to attain the standard FPG level than the other patients at all assessment points (*P* = 0.0654).

### Repaglinide cohort

The association between *PSMD6* rs831571 and clinical features in the repaglinide cohort is shown in Table 2. We found that rs831571 was significantly associated with decreased HbA1c levels after 24 weeks of treatment (*P* = 0.0096). In addition, rs831571 showed significant associations with the homeostatic model assessment of insulin resistance (HOMA-IR) and fasting insulin level after 48 weeks of treatment with repaglinide (*P* = 0.0235 and 0.0212, respectively ). Besides, we also observed there were differences in Δvalues of acute insulin response (AIR) to arginine after 24 weeks of treatment, the decrement of FPG and HbA1c levels after 48 weeks of treatment (*P* = 0.0308, 0.0371 and 0.0101, respectively ).

However, we did not identify any difference in genotype distributions between two groups with different responses to repaglinide, and survival analyses showed no difference between genotype groups.

## Discussion

Rosiglitazone is a member of the TZD drug family, a class of widely used insulin sensitisers that increase insulin-dependent glucose disposal and reduce hepatic glucose output. TZDs act mainly through nuclear receptor peroxisome proliferator-activated receptor-γ (PPARγ). Acting as agonists of PPARγ, TZDs potentiate insulin signaling and improve insulin sensitivity^10^,^11^,^12^. Besides, recent studies reported TZDs could also have its insulin-sensitizing effects independent of PPARγ. For example, it was reported that TZDs could have its effects by activating of AMP-kinase or binding mitochondrial membranes^13^. In addition to improving insulin sensitivity and glycaemic control, rosiglitazone has also been shown to preserve pancreatic ß-cell function and insulin secretion^14^,^15^. Glinides are a type of fasting insulin secretagogue that could help mimic early-phase insulin release, thus providing improved control of postprandial glucose (PPG) levels^16^,^17^. Glinides stimulate insulin secretion by inhibiting ATP-sensitive potassium channels present on the pancreatic β-cell membrane^17^,^18^. Repaglinide is one of most widely used glinides and is effective for lowering PPG and HbA1c^19^,^20^. In addition, HOMA-B and HOMA-IR were significantly improved after repaglinide treatment in clinical trials^21^,^22^,^23^.

The *PSMD6* gene encodes a member of the protease subunit S10 family. The encoded protein is a subunit of the 26S proteasome, which is a member of the UPS, the major cellular machinery responsible for both the recognition and degradation of proteins^7^. UPS is involved in many biological processes, such as protein quality control, cell cycle regulation, gene expression and regulation of life-span, and its alterations are associated with many diseases, including cancer and diabete^24^,^25^,^26^,^27^,^28^. UPS has also been reported to have important roles in mediating the actions of insulin and insulin secretion. Indeed, the inappropriate degradation of proteins involved in the insulin signalling pathway such as the insulin receptor substrate (IRS) by UPS can contribute to the development of insulin resistance^8^,^29^. Furthermore, the role of UPS in insulin secretion has also been identified, and changes in the UPS are involved in human beta cell dysfunction in type 2 diabetes^9^,^30^.

In this study, we observed that *PSMD6* gene variant rs831571 is associated with the efficacy of rosiglitazone and repaglinide. In the rosiglitazone cohort, rs831571 was significantly associated with BMI, FPG, 2-h glucose, and HbA1c levels. HOMA-B was also different among the three genotypes. The rs831571 C allele was significantly associated with better attainment of FPG, and this variant also had an influence on the efficacy of repaglinide on HbA1c and HOMA-IR. As mentioned above, UPS plays important roles in insulin sensitivity and insulin secretion. The activity of UPS was associated with β cell function and insulin section. The downregulation of UPS genes and reduced proteasome activity were observed in T2DM islet^9^. The overactivity of UPS and inappropriate degradation by the UPS of IRS-1 and IRS-2 was associated the development of insulin resistance. Because the 26S proteasome is a component of UPS, SNPs in the *PSMD6* gene encoding this component might have an effect on insulin sensitivity and secretion, thus influencing the effect of oral antidiabetic drugs. Nevertheless, all above are our rational speculation, and the specific underlying mechanism remains to be elucidated in a future functional study.

Several limitations in this study should be noted when interpreting the results. First, the sample size of this study is relatively small, and consequently we may not have had enough statistical power to detect effects of genetic variants on some of the parameters. Second, because we did not adjust for multiple comparisons, we cannot exclude the possibility that our findings were false positive. However, as the variables tested in our study were related, Bonferroni correction was not appropriate and highly conservative. Third, the Kruskal-Wallis test was largely used to analyse the Δ value of parameters among the three genotypes due to the skew distribution; thus, confounding factors such as age, sex, BMI could not be adjusted. Fourth, as the variant of rs831571 is in non-coding area and did not lead to the change of protein function, so we could not carry out the *in vitro* cellular studies to further reveal the underlying molecular mechanism of its pharmacogenomics effects. Fifth, we do not have a control group in this study and we could not exclude the possibility that we are simply observing the effect of genotype and not specifically modification of the response to the medication. So the findings in this study need to be confirmed in the future study with a proper control group.

In conclusion, we observed that the *PSMD6* variant rs831571 might be associated with the therapeutic efficacy of rosiglitazone and repaglinide in Chinese patients with type 2 diabetes. However, in consideration to the limitation of this study and the unknown underlying mechanisms, further studies with a larger sample size and a proper control group as well as functional investigations are necessary in the future.

## Methods

### Patients and study design

All experiments were carried out according to the guidelines and the regulations of the Ethical Committee of Shanghai Jiao Tong University Affiliated Sixth People’s Hospital. The study was approved by the institutional review board of Shanghai Jiao Tong University Affiliated Sixth People’s Hospital, Shanghai, China. Written informed consent was obtained from each patient.

A total of 209 newly diagnosed type 2 diabetes patients, defined according to World Health Organization criteria, were recruited from outpatient clinics in Shanghai, China. Detailed information on this study population has been described previously^3^,^31^,^32^. All patients received no previous pharmacologic therapies for type 2 diabetes prior to the study and were randomly divided into two groups after recruitment. After a 2-week run-in period (diet and exercise therapy only), 104 patients were treated with repaglinide (NovoNorm; Novo Nordisk, Copenhagen, Denmark), and the other 105 patients were given rosiglitazone treatment (Avandia; GlaxoSmithKline, Munich, Germany) for 48 weeks. The patients were visited at weeks 0, 2, 4, 12, 24, 32, and 48, and designed clinical assessments were performed during these visits. Repaglinide was initially administered in a mealtime dosage of 0.5 mg and increased stepwise to 1, 1.5, and 2 mg until the patient achieved a glycaemia target of FPG <7 mmol/L (126 mg/dL) and/or 2 h plasma glucose <11 mmol/L (200 mg/dL). Rosiglitazone treatment was begun with a dosage of 4 mg and titrated to 8 mg when the patient failed to achieve the glycaemic targets. Patients with FPG >13 mmol/L (234 mg/dL), 2hPG >18 mmol/L (324 mg/dL) or HbA1c ≥8% at 2 consecutive time points (a maximal interval of 6 d) were excluded from the study.

### Anthropometric and clinical laboratory measurements

General anthropometric parameters including height (in m), weight (in kg), and systolic and diastolic blood pressure (in mmHg) were measured in all patients at baseline and 48 weeks after repaglinide or rosiglitazone treatment. Body mass index (BMI, kg/m^2^) was calculated as weight/height^2^.

At each visit (weeks 0, 2, 4, 12, 24, 32, and 48), blood samples were collected after an overnight fast and 2 h after a 75 g oral glucose tolerance test (OGTT). Plasma glucose concentrations were measured using the glucose oxidase-peroxidase method with commercial kits (Shanghai Biological Products Institution, Shanghai, China). HbA1c values were determined by high-performance liquid chromatography using a Bio-Rad Variant II haemoglobin testing system (Bio-Rad Laboratories, Hercules, CA, USA) at weeks −2, 0, 12, 24, 32, and 48. Insulin levels in these samples were measured using radioimmunoassay (Linco Research, St Charles, MO, USA).

Arginine stimulation tests were performed to evaluate potential pancreatic beta-cell function on all patients at the beginning of the run-in period (–2 weeks) and 48 weeks after treatment in overnight-fasted states. We collected blood samples at 0 min and 2, 4, and 6 min after intravenous injection of arginine hydrochloride (10% arginine hydrochloride of 50 mL, 5 g) within 30–60 s. The AIR to arginine (in mU/L) was calculated as the mean insulin value of the 2, 4, and 6 min samples minus the fasting insulin concentration.

We used the homeostatic model assessment to estimate insulin resistance and β-cell function^33^. HOMA-IR is calculated using the following formula: fasting insulin concentration (in mU/L) × fasting plasma glucose concentration (in mmol/L)/ 22.5. HOMA-B is calculated using the following formula: 20 × fasting insulin concentration/ (fasting plasma glucose concentration - 3.5).

### Genotyping

Genomic DNA was extracted from peripheral blood leucocytes in whole blood samples. *PSMD6* rs831571 was genotyped by means of matrix-assisted laser desorption/ionisation time-of-flight mass spectroscopy using a MassARRAY platform (MassARRAY Compact Analyser; Sequenom, San Diego, CA, USA). DNA sequencing with a 3130xl Genetic Analyzer (Applied Biosystems, Foster City, CA, USA) was used to confirm the genotyping results.

### Statistical analysis

The data are shown as the mean values ± SEM. Allele frequencies were calculated by gene counting, and Hardy–Weinberg equilibrium tests were performed. Δvalues were calculated as the final value at week 24 or 48 minus the initial value. Multiple linear regressions adjusted for age, gender, BMI and dosage at baseline or signed rank–sum tests were used to assess the differences in quantitative traits among three genotype groups when appropriate. We defined a FPG of <6.1 mmol/L, a 2-h glucose of <7.8 mmol/L, and an HbA1c of <6.5% as attainment. Logistic regression adjusted for age, gender, and BMI at baseline was adopted to estimate genotype distributions between two groups with different responses to the drugs using PLINK (v1.07). The attainment rates between the three genotypes groups were compared by Cox regression model analysis adjusted for age, gender, and BMI at baseline. A two-tailed *P* value of <0.05 was considered statistically significant. The statistical analyses were performed using SAS for Windows (version 8.0; SAS Institute, Cary, NC, USA).

## Additional Information

**How to cite this article**: Chen, M. *et al.* A variant of *PSMD6* is associated with the therapeutic efficacy of oral antidiabetic drugs in Chinese type 2 diabetes patients. *Sci. Rep.* **5**, 10701; doi: 10.1038/srep10701 (2015).

## Figures and Tables

**Table 1:** Association between *PSMD6* rs831571 and clinical features in the rosiglitazone cohort.

**Parameter**		**CC**	**CT**	**TT**	***P* value**
**Age(year)**		50.69 ±1.18	52.91 ±1.52	53.50 ±3.24	0.5793
**Gender(male/female)**		27/8	30/17	7/3	0.3617
**Dosage(mg/day)**		5±0.31	5.87±0.29	5.33±0.67	0.1428
**BMI(kg/m2)**	Baseline	25.03±0.56	24.08±0.45	24.45±0.90	0.8998
	24weeks	24.87±0.69	24.87±0.45	25.56±1.14	**0.0309**
	48weeks	24.77±0.70	24.98±0.47	24.77±1.00	0.2262
	Δ24weeks	–0.38±0.23	0.005±0.18	0.84±0.87	0.0956
	Δ48weeks	–0.29±0.26	0.08±0.20	0.06±0.17	0.2211
**Fasting glucose(mmol/l)**	Baseline	8.80±0.30	9.02±0.27	9.63±0.47	0.2381
	24weeks	6.14±0.19	6.65±0.16	6.74±0.25	**0.0368**
	48weeks	6.47±0.21	6.60±0.19	6.97±0.46	0.4322
	Δ24weeks	–2.63±0.37	–2.38±0.29	–2.96±0.44	0.2826
	Δ48weeks	–2.32±0.33	–2.42±0.32	–3.13±0.49	0.3221
**2-h glucose(mmol/l)**	Baseline	13.74±0.56	12.84±0.43	14.54±0.95	0.9416
	24weeks	7.73±0.45	8.84±0.39	8.87±0.63	**0.0468**
	48weeks	9.02±0.48	8.81±0.33	8.84±0.54	0.9191
	Δ24weeks	–6.17±0.66	–3.99±0.56	–5.90±1.17	**0.0364**
	Δ48weeks	–4.54±0.78	–4.04±0.50	–5.60±1.37	0.1793
**Glycated hemoglobin (%)**	Baseline	8.54±0.26	8.24±0.24	7.96±0.32	0.7197
	24weeks	6.18±0.13	6.59±0.10	6.36±0.17	**0.0233**
	48weeks	6.23±0.15	6.52±0.13	6.19±0.11	0.1179
	Δ24weeks	–2.35±0.26	–1.65±0.24	–1.62±0.30	**0.0247**
	Δ48weeks	–2.25±0.25	–1.54±0.28	–1.79±0.42	**0.0460**
**Fasting insulin (mU/l)**	Baseline	14.15±0.18	15.06±1.18	13.88±1.94	0.9019
	24weeks	16.97±1.67	15.19±1.20	16.95±2.75	0.6440
	48weeks	16.26±1.90	14.90±0.97	22.57±5.10	0.5036
	Δ24weeks	2.51±1.75	0.13±1.34	2.28±2.22	0.7341
	Δ48weeks	1.85±1.61	–0.36±1.35	8.70±5.04	0.1299
**HOMA-IR**	Baseline	5.43±0.42	6.02±0.49	6.01±0.92	0.7522
	24weeks	4.88±0.62	4.44±0.35	5.14±0.90	0.7319
	48weeks	4.82±0.77	5.50±0.38	6.30±1.52	0.7580
	Δ24weeks	–0.66±0.67	–1.58±0.51	–1.26±0.66	0.9670
	Δ48weeks	–0.73±0.65	–1.67±0.55	–0.69±0.97	0.5336
**HOMA-B**	Baseline	61.29±6.32	60.58±6.29	47.71±7.79	0.7316
	24weeks	134.18±9.97	117.53±16.9	106.88±17.0	0.0561
	48weeks	122.55±11.02	108.71±9.14	110.46±15.20	0.4806
	Δ24weeks	71.08±11.08	56.94±16.94	56.26±16.89	0.0547
	Δ48weeks	59.92±11.08	46.54±10.12	60.99±12.52	0.3521
**Acute insulin response(mU/l)**	Baseline	30.28±4.25	32.17±4.08	33.97±3.78	0.4667
	24weeks	27.93±3.90	27.11±2.99	36.04±11.05	0.7830
	48weeks	34.83±5.82	26.53±3.03	36.31±10.28	0.5986
	Δ24weeks	–2.35±3.84	–5.06±3.62	0.91±8.66	0.9454
	Δ48weeks	5.06±5.38	–6.05±4.51	2.34±9.27	0.2180

Note: Data are shown as means ± SEM. *P* values <0.05 are shown in bold. Δ value = T24 or 48 *weeks* value - baseline (T0) value. *P* values were adjusted for age, gender, and BMI at baseline when linear regression model was adopted.

HOMA-B, homeostasis model assessment of β-cell function; HOMA-IR, homeostasis model assessment of insulin resistance.

**Table 2:** Association between *PSMD6* rs831571 and clinical features in the repaglinide cohort.

**Parameter**		**CC**	**CT**	**TT**	***P* value**
**Age(year)**		52.12±1.49	52.52±1.30	54.29±3.98	0.7514
**Gender(male/female)**		19/15	37/13	5/2	0.1311
**Dosage(mg/day)**		2.67±0.27	2.57±0.21	1.53±0.03	0.1692
**BMI(kg/m2)**	Baseline	25.78±0.48	25.16±0.40	25.57±0.66	0.6694
	24weeks	25.61±0.47	25.07±0.44	25.36±0.81	0.5446
	48weeks	25.71±0.48	24.94±0.45	25.69±0.88	0.4215
	Δ24weeks	–0.16±0.16	–0.07±0.20	–0.20±0.22	0.8471
	Δ48weeks	–0.07±0.14	–0.21±0.22	0.13±0.28	0.7226
**Fasting glucose(mmol/l)**	Baseline	9.15±0.31	9.64±0.28	9.39±0.55	0.6672
	24weeks	6.73±0.18	6.39±0.15	6.78±0.47	0.5416
	48weeks	7.18±0.30	6.86±0.19	6.74±0.41	0.9194
	Δ24weeks	–2.39±0.31	–3.30±0.28	–2.61±0.45	0.1695
	Δ48weeks	–1.87±0.32	–2.76±0.29	–2.65±0.53	**0.0371**
**2–h glucose(mmol/l)**	Baseline	13.02±0.55	13.98±0.42	15.33±0.78	**0.0175**
	24weeks	9.34±0.39	8.35±0.29	8.86±0.62	0.1742
	48weeks	9.73±0.56	8.77±0.40	10.05±0.69	0.1555
	Δ24weeks	–3.95±0.70	–5.57±0.50	–6.38±1.04	0.1349
	Δ48weeks	–3.13±0.94	–5.16±0.55	–5.28±1.08	0.3454
**Glycated hemoglobin (%)**	Baseline	7.85±0.19	8.57±0.20	8.48±0.72	0.1006
	24weeks	6.33±0.14	6.15±0.09	5.94±0.12	0.4329
	48weeks	6.44±0.18	6.19±0.10	6.18±0.22	0.8843
	Δ24weeks	–1.51±0.20	–2.42±0.19	–2.54±0.71	**0.0096**
	Δ48weeks	–1.37±0.23	–2.35±0.22	–2.30±0.80	**0.0101**
**Fasting insulin (mU/l)**	Baseline	14.54±1.24	12.49±0.76	13.09±2.31	0.5310
	24weeks	18.91±1.03	17.89±1.18	18.08±2.93	0.4065
	48weeks	19.31±1.16	16.37±1.14	12.29±2.95	**0.0212**
	Δ24weeks	4.36±0.99	5.37±1.10	4.99±3.84	0.9062
	Δ48weeks	4.18±1.20	3.90±1.24	–0.80±2.83	0.2200
**HOMA–IR**	Baseline	5.67±0.43	5.28±0.32	5.52±1.08	0.8258
	24weeks	5.69±0.38	5.07±0.33	5.33±0.78	0.2898
	48weeks	6.10±0.44	4.94±0.36	3.78±0.92	**0.0235**
	Δ24weeks	0.03±0.46	–0.26±0.38	–0.19±1.33	0.7546
	Δ48weeks	0.35±0.47	–0.27±0.45	–1.74±1.11	0.0779
**HOMA–B**	Baseline	62.66±12.46	46.41±4.20	45.93±8.61	0.5175
	24weeks	128.00±9.08	149.51±15.04	128.37±29.23	0.9195
	48weeks	126.24±14.97	117.70±12.38	77.43±19	0.1525
	Δ24weeks	63.70±12.32	103.08±14.16	82.43±30	0.5251
	Δ48weeks	60.97±15.16	70.58±11.72	31.49±17.12	0.3893
**Acute insulin response(mU/l)**	Baseline	31.92±3.15	24.92±3.01	28.79±7.20	0.1084
	24weeks	27.81±3.08	28.11±3.07	20.16±6.44	0.7199
	48weeks	29.33±3.32	24.36±2.71	28.31±8.31	0.3450
	Δ24weeks	–4.10±3.40	3.95±2.58	–8.63±5.93	**0.0308**
	Δ48weeks	–3.31±4.36	–0.66±2.30	–0.47±7.69	0.9131

Note: Data are shown as means ± SEM. *P* values <0.05 are shown in bold. Δ value = T24 or 48 *weeks* value - baseline (T0) value. *P* values were adjusted for age, gender, and BMI at baseline when linear regression model was adopted.

HOMA-B, homeostasis model assessment of β-cell function; HOMA-IR, homeostasis model assessment of insulin resistance.
